# Two cases of relapsed HIV-associated visceral leishmaniasis successfully treated with combination therapy

**DOI:** 10.1186/s12981-018-0215-x

**Published:** 2018-12-20

**Authors:** Antonio Mastroianni, Paolo Gaibani, Giada Rossini, Caterina Vocale, Maria Carla Re, Gianfranco Ravaglia, Vittorio Sambri, Stefania Varani

**Affiliations:** 1 Unit of Infectious and Tropical Diseases, St. Annunziata Hospital, Cosenza, Italy; 2 Unit of Microbiology, Regional Reference Centre for Microbiological Emergencies (CRREM), St. Orsola-Malpighi University Hospital, Bologna, Italy; 3 Department of Experimental, Diagnostic and Specialty Medicine, University of Bologna, Bologna, Italy; 4 Pharmacy Unit, “G.B. Morgagni-L. Pierantoni” Hospital, Forlì, Italy; 5 Unit of Microbiology, the Greater Romagna Area Hub Laboratory, Pievesestina (Forlì-Cesena), Italy

**Keywords:** Opportunistic infection, Protozoal infection, Visceral leishmaniasis, Disseminated cutaneous leishmaniasis

## Abstract

**Background:**

The management of visceral leishmaniasis (VL) in HIV-infected patients is often complex with patients experiencing higher mortality rates, more toxic side effects and a higher possibility of treatment failure and relapse than HIV-negative individuals with VL.

**Case presentation:**

We report on successful salvage therapy in two HIV-infected patients suffering with disseminated cutaneous and visceral leishmaniasis, recalcitrant to therapy with liposomal amphotericin B. After the employment of combination anti-leishmanial treatment, parasite genomes were not detectable up to the last follow up visit, 57 and 78 weeks after treatment onset, respectively. CD4+ lymphocyte counts fluctuated over time, but were generally higher than counts detected at treatment onset, which likely contributed to protection against VL relapse.

**Conclusions:**

Results achieved with the anti-leishmanial combination treatment were promising, but are based on only two patients. Future investigation is necessary to confirm the efficacy of this salvage therapy in sustaining the immunological response and control of VL.

## Background

Visceral leishmaniasis (VL) and HIV co-infection is common in tropical areas and can occur in southern European countries [1]. Concerning the WHO European Region, the estimated annual incidence of VL/HIV co-infection is around 1100–1900 cases/year [2]. The *Leishmania*-HIV interplay at the cellular and molecular level appears to affect the course of infection for both pathogens [3] as VL hampers the immunological competence of HIV-positive patients, while HIV-infected patients with VL exhibit reduced anti-leishmanial drug response, frequent relapses and higher mortality rates than HIV-negative individuals [1, 2]. A combination anti-leishmanial therapy might be considered in HIV/VL co-infection, especially for HIV patients with refractory cases of VL [4, 5]. Nevertheless, a valuable multidrug therapy and optimal treatment duration have yet to be established.

## Case description

We report on two patients with *Leishmania infantum*-induced VL and HIV-1 co-infection who received an extended course of a salvage therapy (anti-leishmanial combination treatment, cAnti-Leish) in addition to antiretroviral therapy (ART). The rationale behind multidrug therapy against *Leishmania* was to increase drug activity with compounds targeting different pathways of the parasite metabolism, ultimately preventing the emergence of drug resistance. Drugs with distinct activity against *Leishmania* were used, including those targeting ergosterol biosynthesis (liposomal amphotericin B [L-amB] and fluconazole) and cell membrane integrity (L-amB), modulating surface signaling pathways (miltefosine) interfering with the purine salvage pathway (allopurinol) and hindering DNA synthesis at mitochondrial level (pentamidine) [6, 7]. Furthermore, HIV-1 protease inhibitors (PI) were employed as adjunctive anti-leishmanial therapy for HIV-associated VL [8]. In fact, in vitro [9] and in vivo [10] evidence suggests that high dose PI hamper *Leishmania* replication and exhibit synergistic effect with L-amB and miltefosine [10].

Our regimen resulted in good control of VL and HIV-1 infection in both patients, whereas previously administered conventional monotherapy with L-amB (dosage regimen of 4 mg/kg on days 1–5, 10, 17, 24, 31 and 38, for a total dose of 40 mg/kg) followed by secondary prophylaxis (3 mg/kg every 4 weeks) did not prevent multiple VL relapses. The study was conducted in accordance with the Declaration of Helsinki. Written informed consent was obtained from the patients included in the study.

Case 1 was a 55-year-old man who presented at the Unit of Infectious Diseases, Forlì Hospital (northeastern Italy) with a 4-year history of HIV infection and a 2-year history of disseminated cutaneous leishmaniasis. Despite being under secondary prophylaxis with L-amB, severe gastroenterological disease caused by *Leishmania* was discovered. Case 2 was a 50-year old HIV-positive man who presented with a 6-year history of HIV infection and a 3-year history of disseminated cutaneous leishmaniasis and VL, recalcitrant to therapy with L-amB.

**Fig. 1:**
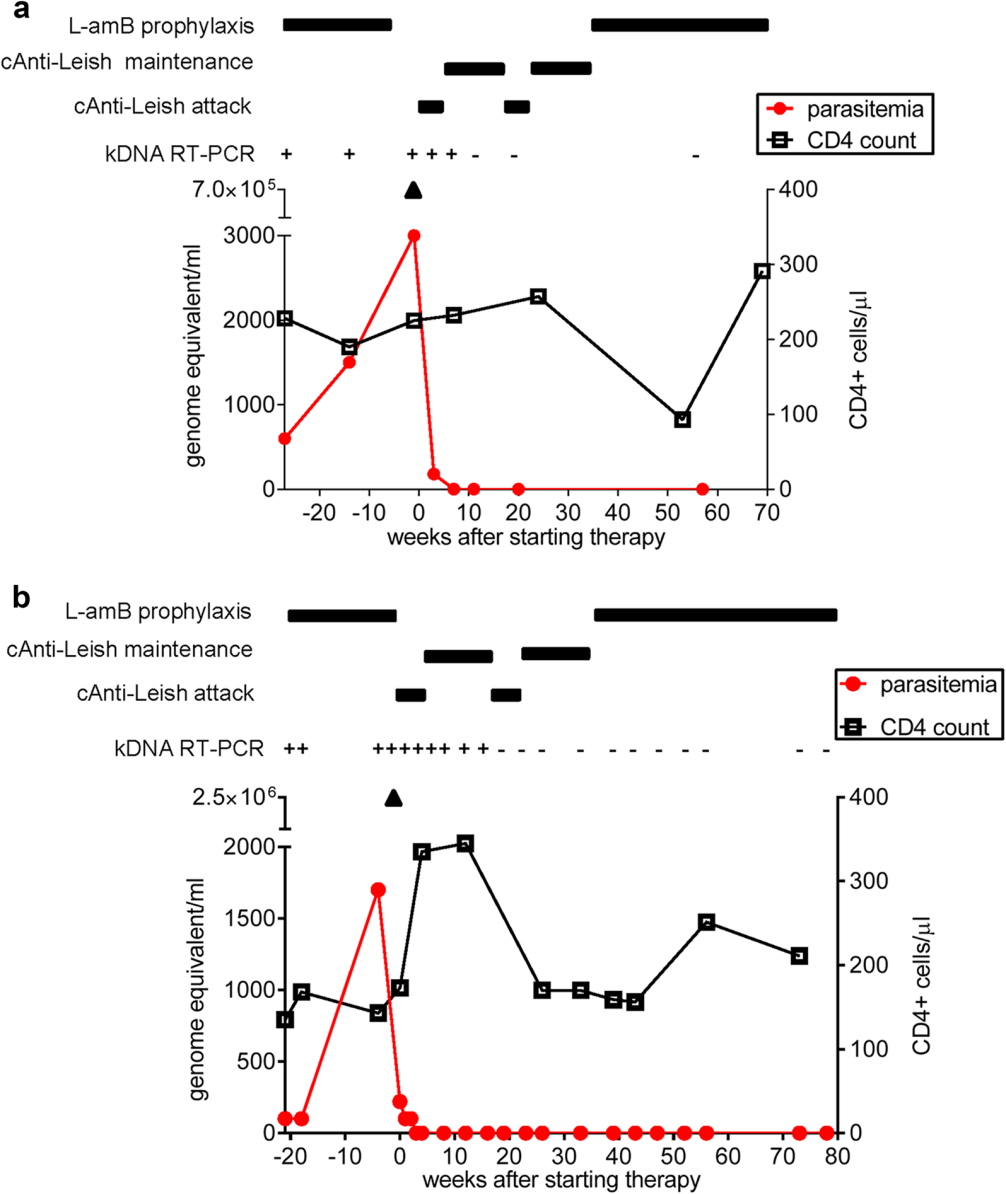
Time course of parasitological and immunological parameters upon combined anti-leishmanial treatment in Case 1 (**a**) and Case 2 (**b**). Parasitemia (red circles) was measured by quantitative real-time PCR (qPCR) before and after anti-leishmanial combination (cAnti-Leish) treatment. Also shown is the CD4+ cell count in peripheral blood (empty squares; normal range: 500–1500 cells/μL). HIV RNA was < 40 copies/mL in Case 1 and Case 2 throughout the study as tested by the Veris DxN HIV-1 assay (Beckman Coulter, Brea, CA USA). Leishmanial DNA was detected in peripheral blood by two distinct real-time (RT) PCR assays, amplifying segments of the small-subunit ribosomal RNA gene and the kinetoplast DNA (kDNA) [15]. Samples testing positive for parasitic DNA were then quantified by RT-PCR for the single copy polymerase gene (*pol*) of *Leishmania* [16]. The detection limit was 0.00005 parasite/reaction for kDNA RT-PCR and 1 parasite/reaction for *pol* RT-PCR. *Leishmania* typing was performed by hsp70 PCR [17] and *L. infantum* was identified in both patients. ▲: Parasitemia was quantified in bone marrow aspirates within one week before cAnti-Leish onset

Leishmanial DNA was detected by real-time PCR in the peripheral blood, bone marrow aspirates and skin biopsies of both patients (Fig. 1 and not shown). Additionally, stomach and colon biopsies from Case 1 tested positive for leishmanial DNA. In both cases, bone marrow showed high levels of parasitemia (around 10E6 parasite genomes/mL), while lower levels (around 10E2–10E3 parasite genomes/mL) were detected in peripheral blood (Fig. 1). Patients exhibited a CD4+ count of 225 cells/μL and 170 cells/μL before cAnti-Leish, Case 1 and Case 2, respectively. The nadir CD4 cell count was 120 cells/μL and 143 cells/μL, Case 1 and Case 2, respectively.

High and intermittent doses of L-amB in 5% dextrose i.v. infusion over 4 h (4 mg/kg/day on days 1–5, 10, 17, 24, 32 and 38) were administered for VL treatment [1, 4]. The patients also received concomitant miltefosine (50 mg three times/day for 28 days), allopurinol (300 mg four times daily for 15 days) and de-escalating doses of fluconazole (from 600 to 400 mg/day for 28 days). Since PI can also be considered an adjunctive anti-leishmanial therapy, ART was modified by adding a boosted PI treatment, including darunavir (800 mg/day) and ritonavir (100 mg/day) in Case 1 and atazanavir (300 mg/day) and ritonavir (100 mg/day) in Case 2, respectively, while the backbone therapy included lamivudine 300 mg/day and tenofovir disoproxil fumarate (245 mg/day) in both patients. Once remission was achieved, including resolution of fever, remarkable improvement of the patient’s general condition, regression of splenomegaly and amelioration of laboratory parameters, a maintenance treatment was instituted for 12 weeks with L-AmB (2–3 mg/kg every 3 weeks), miltefosine (50 mg three times weekly), allopurinol (300 mg twice daily), fluconazole (400 mg/day) and pentamidine aerosol (300 mg twice a month for the first 2 months, then once a month for 6 months). One week after cAnti-Leish onset, there was a significant decline in peripheral blood parasitemia (Fig. 1); we observed a good correlation between clinical outcome and blood parasite burden. To consolidate activity against potentially residual parasites, a second cycle of the initial and maintenance therapy was given to both patients (Case 1 only received 2 mg/kg L-Amb in the second cycle). Secondary prophylaxis with L-Amb (2–3 mg/kg every 3–4 weeks) was subsequently administered. Parasite genomes were not detectable over time up to the last follow-up visit (Week 57 and Week 78 for Case 1 and Case 2, respectively). CD4+ lymphocyte counts fluctuated over time, but were generally higher than counts detected at treatment onset, which likely contributed to protection against VL relapse.

## Discussion and conclusions

It has been suggested that combined drug regimens are the best option for VL/HIV co-infected patients by increasing drug efficacy, reducing toxicity and hampering the emergence of resistance [4, 5]. So far, the best combination therapy appears to be L-amB plus miltefosine due to the observed synergism between the two compounds in vitro and in animal models [11]. Furthermore, a combination regimen consisting of L-amB and miltefosine was employed in VL/HIV co-infected patients in a pilot study performed by Médecin Sans Frontières in Ethiopia [12] as well as in 120 VL/HIV co-infected patients in India [13], showing encouraging results.

Here, the employment of L-amB and miltefosine was associated to other drugs with anti-leishmanial effects, including fluconazole, allopurinol and pentamidine. Results achieved with cAnti-Leish were promising, but are based on only two patients. Thus, future investigation is necessary to confirm the efficacy of this salvage therapy. Furthermore, the potentially synergistic role of cAnti-Leish and a PI-based ART regimen in sustaining the immunological response and control of VL should be further explored as recent evidence suggests that both CD4+ count and circulating *Leishmania* DNA contribute to prognosis in HIV/VL co-infection [14].
